# Time Course of Odor Categorization Processing

**DOI:** 10.1093/texcom/tgab058

**Published:** 2021-10-05

**Authors:** Jisub Bae, Kwangsu Kim, Sun Ae Moon, Han Kyoung Choe, Youngsun Jin, Won-Seok Kang, Cheil Moon

**Affiliations:** Brain Engineering Convergence Research Center, Daegu Gyeungbuk Institute of Science and Technology (DGIST), Daegu, South Korea; Department of Brain & Cognitive Sciences, Daegu Gyeungbuk Institute of Science and Technology (DGIST), Daegu, South Korea; Convergence Research Advanced Centre for Olfaction, Daegu Gyeungbuk Institute of Science and Technology (DGIST), Daegu, South Korea; Department of Brain & Cognitive Sciences, Daegu Gyeungbuk Institute of Science and Technology (DGIST), Daegu, South Korea; Department of Brain & Cognitive Sciences, Daegu Gyeungbuk Institute of Science and Technology (DGIST), Daegu, South Korea; Convergence Research Advanced Centre for Olfaction, Daegu Gyeungbuk Institute of Science and Technology (DGIST), Daegu, South Korea; Department of Psychology, Kyungpook National University, Daegu, South Korea; Convergence Research Advanced Centre for Olfaction, Daegu Gyeungbuk Institute of Science and Technology (DGIST), Daegu, South Korea; Division of Intelligent Robot, Daegu Gyeungbuk Institute of Science and Technology (DGIST), Daegu, South Korea; Department of Brain & Cognitive Sciences, Daegu Gyeungbuk Institute of Science and Technology (DGIST), Daegu, South Korea; Convergence Research Advanced Centre for Olfaction, Daegu Gyeungbuk Institute of Science and Technology (DGIST), Daegu, South Korea

**Keywords:** EEG, gamma, odor categorizing, odor quality, olfactory processing, olfactory system, theta, time course

## Abstract

The brain’s mechanisms for categorizing different odors have long been a research focus. Previous studies suggest that odor categorization may involve multiple neurological processes within the brain with temporal and spatial neuronal activation. However, there is limited evidence regarding temporally mediated mechanisms in humans, especially millisecond odor processing. Such mechanisms may be important because different brain areas may play different roles at a particular activation time during sensory processing. Here, we focused on how the brain categorizes odors at specific time intervals. Using multivariate electroencephalography (EEG) analysis, we found that similarly perceived odors induced similar EEG signals during 50–100, 150–200, and 350–400 ms at the theta frequency. We also found significant activation at 100–150 and 350–400 ms at the gamma frequency. At these two frequencies, significant activation was observed in some olfactory-associated areas, including the orbitofrontal cortex. Our findings provide essential evidence that specific periods may be related to odor quality processing during central olfactory processing.

## Introduction

The brain consistently classifies complex external stimuli. However, our perception of odor changes over time, even when the same olfactory stimuli persist. Olfactory perception is affected not only by external properties, such as the odor’s physicochemical features, but also by internal properties, such as memory during olfaction (Olofsson et al. 2013). We are still far from fully understanding the temporal changes of olfactory perception and their neural mechanisms, so it is important to clarify the temporal dynamics of odor categorization in the human brain.

Various studies have provided essential evidence of how the brain categorizes odors (Sobel et al. 1998; Spors and Grinvald 2002; Rennaker et al. 2007; Su et al. 2011). They suggested that an odor can be categorized by temporal- and spatial scale-specific neuronal activity. Distinguishable temporal distribution of odor-evoked activity in the piriform cortex (PC) has been induced by different odorants (Rennaker et al. 2007). Subsequent evidence suggests that population coding of PC neurons may be a key in the odor categorizing mechanism (Miura et al. 2012). Moreover, specific frequencies (e.g., beta and gamma) may be related to the odor categorizing process and have displayed different neuronal patterns following different olfactory input (Martin and Ravel 2014). It has also been demonstrated that spatial neuronal activity in the PC encodes every detail of different odors (Schoenbaum and Eichenbaum 1995; Kadohisa and Wilson 2006; Yoshida and Mori 2007). Increasing evidence from rodent studies indicates the importance of the orbitofrontal cortex (OFC) in mediating odor categorization processing (Schoenbaum and Eichenbaum 1995; Schoenbaum et al. 2002). These studies provide precise information relating to how the brain categorizes odor.

Human studies on odor categorization have also been conducted, but they are more biased toward spatial evidence. Increasing evidence indicates the importance of the PC in human olfactory stimulation (Zald and Pardo 2000; Wilson and Sullivan 2003; Gottfried et al. 2006) and suggests that, together with the OFC, it may mediate odor categorization (Zald and Pardo 2000; Gottfried 2010). Some studies have suggested that systemic-phase synchronization may be related to odor categorization because it varies depending on the odor used for stimulation (Klemm et al. 1992; Lorig and Schwartz 2013) and the theta burst in the PC (Jiang et al. 2017). To date, there is little evidence for the role of temporal encoding in humans. Some studies characterizing temporal activities during odor processing suggest that olfactory-related areas are activated from around 50 or 80 ms (Masaoka et al. 2014; Stadlbauer et al. 2016), and olfactory-specific activation of the PC starts at 110 ms (Jiang et al. 2017) in the olfactory bulb (Iravani et al. 2020). However, when and how odor categorization occurs during olfactory processing requires elucidation. This issue may be more important in human studies because functional MRI (fMRI) or positron emission tomography usually shows results accumulated over periods longer than 1 s.

Thus, we studied when and how the human brain categorizes odor quality during olfactory processing. First, we determined time points when a pair of similar odors were categorized as a close distance by classification analysis. Second, we focused on the relation between these determined time points and olfactory-associated areas. We used two odorants, 2-acetylpyrazine (AP) and 2, 3, 5-trimethyl pyrazine (TP), which are described as similar to each other by humans (TGSC database, www.thegoodscentscompany.com). Hexan-1-al (HA) was used as a control odor and was considered distinct from AP and TP. Electroencephalography (EEG) was used to measure the temporal resolution in the time period of interest. We focused on the 0–400 ms time frame because behavioral evidence suggests that odor discrimination occurs within 420 ms of odor stimulation (Laing 1986). Because sufficient evidence is available from time-frequency-based studies across rodent species, we performed event-related spectral perturbation (ERSP) analysis using the multivariate method to link with animal studies. ERSPs were analyzed in theta, alpha, beta, and gamma frequency bands since each are reportedly indicative of various olfaction processes. Theta waves have been reported to be altered when distinct odors are used for stimulation (Klemm et al. 1992; Lorig and Schwartz 2013). Theta waves are associated with olfactory-associated areas, such as the hippocampus (Ekstrom et al. 2005; Montgomery et al. 2009) and PC (Jiang et al. 2017). Alpha waves may play a role in odor valence (Kline et al. 2000). Beta and gamma waves are reportedly critical for odor-information processing in rodents (Adrian 1942; Bressler 1984; Vanderwolf 1992; Chapman et al. 1998; Kay and Freeman 1998; Mori et al. 2013) and humans (Aydemir 2017; Iravani et al. 2020). However, odor categorization evidence from EEG studies is still lacking. Here, we performed multivariate analysis rather than directly compared a direct ERSP signal to solve a question. Because spatial and temporal neural representations play critical roles in the olfactory network (Laurent et al. 1996; Spors and Grinvald 2002; Wang et al. 2003), we performed standardized low-resolution brain electromagnetic tomography (sLORETA) analysis to evaluate the activities of olfactory-associated areas (Pascual-Marqui 2002).

## Materials and Methods

### Participants

After giving informed consent, 24 participants were included in the experiment (15 females, 9 males; mean age 19 years [SD = 2.37]; all participants were right-handed, displayed normal olfactory functions, and had no history of psychologic or neurologic diseases). The study was approved by the Institutional Review Board ethics committee, Daegu Gyeongbuk Institute of Science & Technology (DGIST-150709-HR-014-01).

### Odor Preparation and Delivery

**Figure 1:**
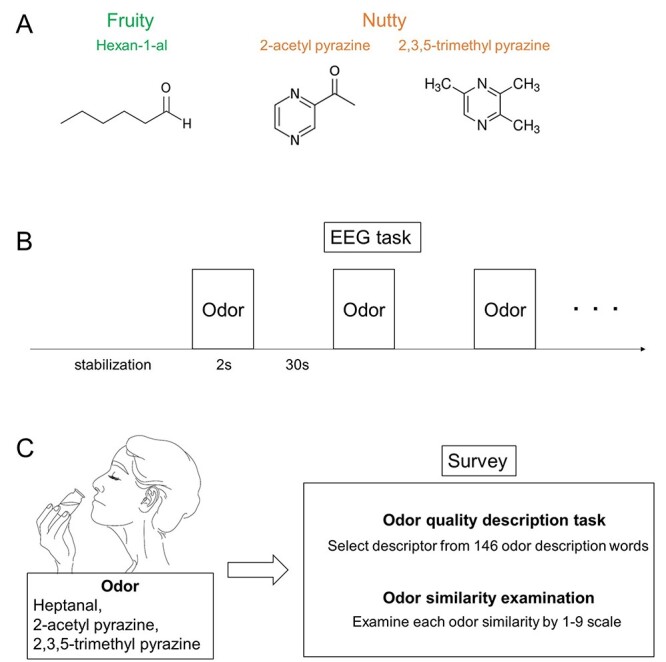
Experimental scheme. (*A)* Odor stimulation. (*B*) EEG experimental procedure: a stabilization period (60 s) was followed by stimulation (2 s) and rest (30 s). During the rest period, fixation was performed. (*C*) Survey procedure.

2-Acetlypyrazine (Sigma-Aldrich, LOT#MKCB1629V), 2,3,5-trimethylpyrazine (Sigma-Aldrich, LOT#STBG6509), and hexan-1-al (Sigma-Aldrich, LOT#MKCC2925) were used as olfactory stimuli (Fig. 1*A*). 2-Acetlypyrazine and 2,3,5-trimethylpyrazine were each diluted in distilled water (DW) to a concentration of 0.1%. Hexan-1-al was diluted in polyethylene glycol (Sigma-Aldrich, LOT#BCBP4448V) to a concentration of 0.5%. DW was used for baselining. We set the odor concentration by adjusting odor intensity to approximately 5 (moderate intensity). A respiration sensor was used for odor delivery (Accessory of ActiveTwo, BioSemi, Amsterdam, the Netherlands). Odors were delivered using a custom-built olfactometer with a nasal mask (airflow 3.90 l/min). Odor delivery was initiated during exhalation and terminated 2 s after the inhalation starting point (Supplementary Fig. 1).

### Electroencephalogram Recording

EEG signals were recorded with and without odor stimulation and digitalized via an EEG amplifier (ActiveTwo, BioSemi). Data were sampled at 2048 Hz and analogue-filtered via a 0.15-Hz high-pass filter and a 100-Hz low-pass filter. Additionally, a notch filter at 50 Hz was applied. EEG signals were recorded from 64 channels using Ag/AgCl scalp electrodes arranged according to the extended 10–20 system and mounted on a cap (headcap 64 ch, BioSemi) at the following sites: Fp1, AF7, AF3, F1, F3, F5, F7, FT7, FC5, FC3, FC1, C1, C3, C5, T7 (T3), TP7, CP5, CP3, CP1, P1, P3, P5, P7, P9, PO7, PO3, O1, Iz (inion), Oz, POz, Pz, CPz, Fpz, Fp2, AF8, AF4, Afz, Fz, F2, F4, F6, F8, FT8, FC6, FC4, FC2, FCz, Cz, C2, C4, C6, T8 (T4), TP8, CP6, CP4, CP2, P2, P4, P6, P8, P10, PO8, PO4, and O2. Impedances were kept below 15 kΩ. The ocular activity was measured via EOG channels mounted at the right eye’s outer canthi and approximately 2 cm above and below the right eye.

### E‌EG Preprocessing

First, the data were downsampled to 512 Hz. Next, each of the 64 EEG channels was filtered off-line with high- and low-pass filters set to 0.5 and 50 Hz, respectively, to exclude noise caused by skin potential, the DC component of the amplifier, and muscle artifacts. Data were segmented into 1500-ms epochs, each ranging from −500 to 1000 ms relative to the inhalation starting point. The filtered EEG channels were referenced to an average of all electrodes. Individual subject data were visually inspected for eye and muscle artifacts prior to artifact removal via the automatic artifact rejection procedure, which is described below:

(1) Epoch rejection: epochs were rejected if their amplitude outranged −50 to 50 μV after ocular artifact correction (Gratton et al. 1983).
(2) Channel rejection: channels were rejected if the remaining epochs were less than 25 trials after epoch rejection.
(3) Recording rejection: a participant was excluded if they had 4 or more rejected trials.

### Experimental Paradigm of EEG Recording

Odor stimuli were presented during exhalation, and the inspiration starting point was marked for time-locking analysis (Supplementary Fig. 1). Before stimuli, a white cross was displayed on the LCD monitor; a blank screen (i.e., black background) was displayed during odor stimulation. The room humidity was < 15%–60%, and the temperature was maintained between 21°C and 25°C to ensure a fixed vapor pressure of the odor. The experimental room was fitted with soundproof walls and ventilation facilities to reduce noise and limit unexpected sensory stimuli. To reduce these effects from uncontrolled factors, we presented odor as a random sequence, and measured EEG separately over three days per participants, who were blinded.

Before the experiments, we explained about the devices we used and informed that odor or air might randomly come through the mask. We instructed participants to relax to ensure a high-quality respiration cycle. During EEG recording, participants were seated upright in a chair and instructed to fix their gaze on the center of the LCD monitor. A stabilization period (60 s) was used to ensure that participants were relaxed (Fig. 1*B*), followed by odor stimulation with an olfactometer for 2 s during one inspiration. A 30-s rest period was used between odor stimulations to avoid odor adaptation; rest periods consisted of “Blank” (black background presented) and “Fixation” (white cross-displayed) sessions. ‘The first 20 s of the rest period were set as “Blank,” the next 9 s as “Fixation,” and the last 1 s as “Blank” again. All experiments included at least 30 trials per condition.

### Multivariate Analysis

#### E‌EG Data Extraction for Analysis

For the multivariate analyses, the data were extracted from raw EEG data sets. For time-variant data, ERSP was extracted from each of the 64 electrodes. ERSPs were obtained by wavelet transformation of the preprocessed EEG signals in EEGLAB software (Delorme and Makeig 2004) at 100 log-spaced frequencies ranging from 0.5 to 50 Hz. The resulting ERSPs ranged from −220 to 720 ms (200 time points). The wavelet cycle used 1 and 25 cycles as the lowest and highest frequencies, respectively. The baseline was set as the entire pre-stimulation period (before 0 ms). DW was used for baselining to reduce olfactory-associated signals. Each ERSP was divided into four data sets corresponding to the following frequency bands: 4–8 Hz (theta), 8–13 Hz (alpha), 13–30 Hz (beta), and 30–50 Hz (gamma). Each of the four data sets was divided into eight subsets: 0–50, 50–100, 100–150, 150–200, 200–250, 250–300, 300–350, and 350–400 ms. Each subset was averaged by frequency before multivariate analysis. Therefore, the extracted data contained the average value of each frequency band for each time point and electrode.

(1) \begin{equation*} {\boldsymbol{f}}_{\boldsymbol{CHn}-\boldsymbol{Tm}}\left[\boldsymbol{l}\right]=\frac{\mathbf{1}}{\boldsymbol{N}}{\sum}_{\boldsymbol{l}}^{\boldsymbol{l}+\boldsymbol{N}-\mathbf{1}}{\boldsymbol{ERSP}}_{\boldsymbol{CHn}-\boldsymbol{Tm}}\left[\boldsymbol{l}\right] \end{equation*}

where ***T*m** is the ERSP time point, ***CH*n** is the EEG electrode number, $\boldsymbol{l}$ is the start point, and $\boldsymbol{l}+\boldsymbol{N}-\mathbf{1}$ is the endpoint of a specific frequency band (i.e., theta, alpha, beta, or gamma). For example, ${\boldsymbol{ERSP}}_{\boldsymbol{C}H1-\boldsymbol{T}1}\Big[\mathbf{3}\Big]$ means “third frequency point ERSP value of electrode channel 1 and time point 1” and ${\boldsymbol{f}}_{\boldsymbol{CH}\mathbf{1}-\boldsymbol{T}\mathbf{1}}$ means “average ERSP value of electrode channel 1 and time point 1.” There was a total of 11 ***T*m**s for each time window. These average ERSP values from each electrode channel and time point were concatenated to form the EEG-feature vector that varied with time:

(2) \begin{eqnarray*}&& {\boldsymbol{F}}_{\boldsymbol{Odor}-\boldsymbol{frequency}\ \boldsymbol{band}-\boldsymbol{T}-\boldsymbol{P}}\nonumber\\&&={[{\boldsymbol{f}}_{\boldsymbol{CH}\mathbf{1}-\boldsymbol{T}\mathbf{1}}\left[\boldsymbol{l}\right],{\boldsymbol{f}}_{\boldsymbol{CH}\mathbf{1}-\boldsymbol{T}\mathbf{2}}\left[\boldsymbol{l}\right],{\boldsymbol{f}}_{\boldsymbol{CH}\mathbf{1}-\boldsymbol{T}\mathbf{3}}\left[\boldsymbol{l}\right]\dots \dots, {\boldsymbol{f}}_{\boldsymbol{CH}\boldsymbol{n}-\boldsymbol{T}\boldsymbol{m}}\left[\boldsymbol{l}\right]]}_{\boldsymbol{P}} \end{eqnarray*}

where ***Odor*** is the odor condition (i.e., HA, AP, TP), ***frequency band*** is the EEG frequency band (i.e., theta, alpha, beta, gamma), ***T*** is the time window (i.e., 0–50 ms, 50–100 ms, 100–150 ms, 150–200 ms, 200–250 ms, 250–300 ms, 300–350 ms, 350–400 ms), ***P*** is the participant, ${\boldsymbol{F}}_{\boldsymbol{Odor}-\boldsymbol{frequency}\ \boldsymbol{band}-\boldsymbol{T}-\boldsymbol{P}}$ signifies “extracted ERSP data of each frequency band, time window, and odor condition from each participant.”

#### Classification Design and Procedure

Following EEG data extraction, 576 vectors (3 odors × 1 frequency band × 24 participants × 8 time windows) were extracted from ERSP. Each vector represented the spatiotemporal activity of EEG containing odor information. Because we focused on the induction of similar brain activities by different odors in view of specific frequency bands and time points, a single data set was ${\boldsymbol{F}}_{\boldsymbol{Odor}-\boldsymbol{frequency}\ \boldsymbol{band}-\boldsymbol{T}-\boldsymbol{P}}$ while $\boldsymbol{frequency}\ \boldsymbol{band}$ and $\boldsymbol{T}$ were fixed.

**Figure 2:**
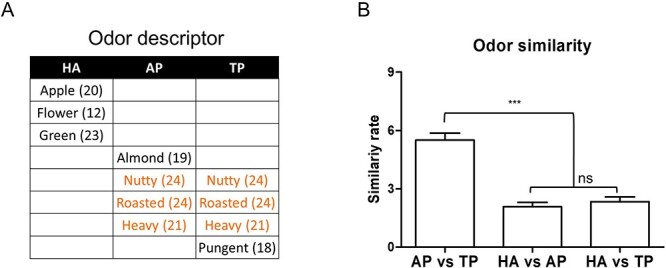
Similarities between odors. (*A*) Quality descriptors for each odor selected by at least 12 participants after odor stimulation. Orange indicates the same descriptor chosen for AP and TP. (*B*) Results of the odor similarity survey. The similarity between AP and TP was significantly higher than that between AP and HA and between TP and HA.

Using these extracted data sets, Library for Support Vector Machines (LIBSVM, https://www.csie.ntu.edu.tw/~cjlin/libsvm/) was used to decode odor information and quantify the similarity between odors. The SVM classifier was trained on ERSP patterns using a pair of odors (AP vs. TP; HA vs. TP; HA vs. AP) (Supplementary Fig. 2*A*, middle panel). The model was verified using the training data set. Next, the classifier was tested on the ERSP of the odor (Supplementary Fig. 2*A*, right panel). Because this analysis is based on odor object quality rather than odor valence or intensity (Fig. 2 and Supplementary Fig. 3), the EEG signal may provide information on the object quality of each specific odor. To verify whether each odor was classified within the appropriate class, we also performed an odor similarity verification procedure (Supplementary Fig. 2*B*). When two odors were identically classified by the procedure outlined in Supplementary Figure 2*B* (left panel), these two odors were considered similar. When the odor was not classified as similar to both odors in the training odor pair, this odor was defined as not similar to either of the training odors.

### sLORETA Imaging

sLORETA (Pascual-Marqui 2002) was used to estimate the intracerebral electrical sources that generated the scalp-recorded activity in theta frequency bands. sLORETA computes electric neuronal activity as current density (A/m^2^) without assuming a predefined number of active sources. We used regions of interest (ROI) in all Brodmann areas (BA). Time-varying cross-spectrum data were obtained by ROI analysis.

### Statistical Analysis

Results are shown as mean ± SEM and the significance threshold was set at *P* < 0.05 (*P < 0.05, **P < 0.01, ***P < 0.001). To verify the rates of odor responses determined by surveying the participants, Friedman one-way analysis of variance (ANOVA) was performed. To verify column differences, the Bonferroni post hoc test was used.

### Survey

We measured “intensity” and “hedonicity” using rating scales of 1–9 to measure the odor response. To quantify the perceived similarities between odors, the participants performed an odor quality description task and similarity examination. For the former, following odor stimulation, the participants chose suitable odor quality descriptions from 146 odor descriptions (Dravnieks 1992). To assess similarities, the participants used a similarity scale (between 1 and 9, modified from (Engen 1964)).

### Software

Electrophysiological data were analyzed using MATLAB 2016b, in conjunction with the toolboxes EEGLAB (Delorme and Makeig 2004) and LibSVM (Chang and Lin 2011). MATLAB was also used for statistical analyses.

## Results

### AP and TP Show Similar Odor Object Quality

Because odor object quality and EEG signal can be influenced by intensity and hedonicity, we first confirmed no differences in odor intensity or hedonicity among the odors tested (Supplementary Fig. 3). No significant differences were observed between participant-rated odors in intensity (X^2^ [2, 71] = 0.67, *P* = 0.71, Friedman one-way ANOVA) or hedonicity (X^2^ [2, 71] = 2.48, *P* = 0.29, Friedman one-way ANOVA).

To verify odor similarity, the participants were asked to select specific descriptors of AP, TP, and HA odors (Fig. 2*A*). Similar descriptors (e.g., “nutty,” “roasted,” and “heavy”) were chosen for AP and TP, but those used to describe HA were dissimilar. Next, we performed a similarity survey to verify the similarity between odors (Fig. 2*B*; X^2^ [2, 71] = 32.00, *P* < 0.0001, Friedman one-way ANOVA). Participants rated the similarity of two selected odors using a 1-to-9 scale (larger numbers reflect more similarity). Consistent with the odor descriptor results, we found that AP and TP were more similar to each other than odors in other combinations (*P* < 0.0001, Bonferroni post hoc test).

### Multivariate Patterns of Theta and Gamma ERSPs Indicate that AP and TP Induced Similar ERSPs at Specific Time Intervals

To understand the ERSP signals representing temporal and frequency similarities between odors, we ensured that similar multivariate patterns of brain signals were induced by odor stimuli (see section Multivariate Analysis).

**Figure 3:**
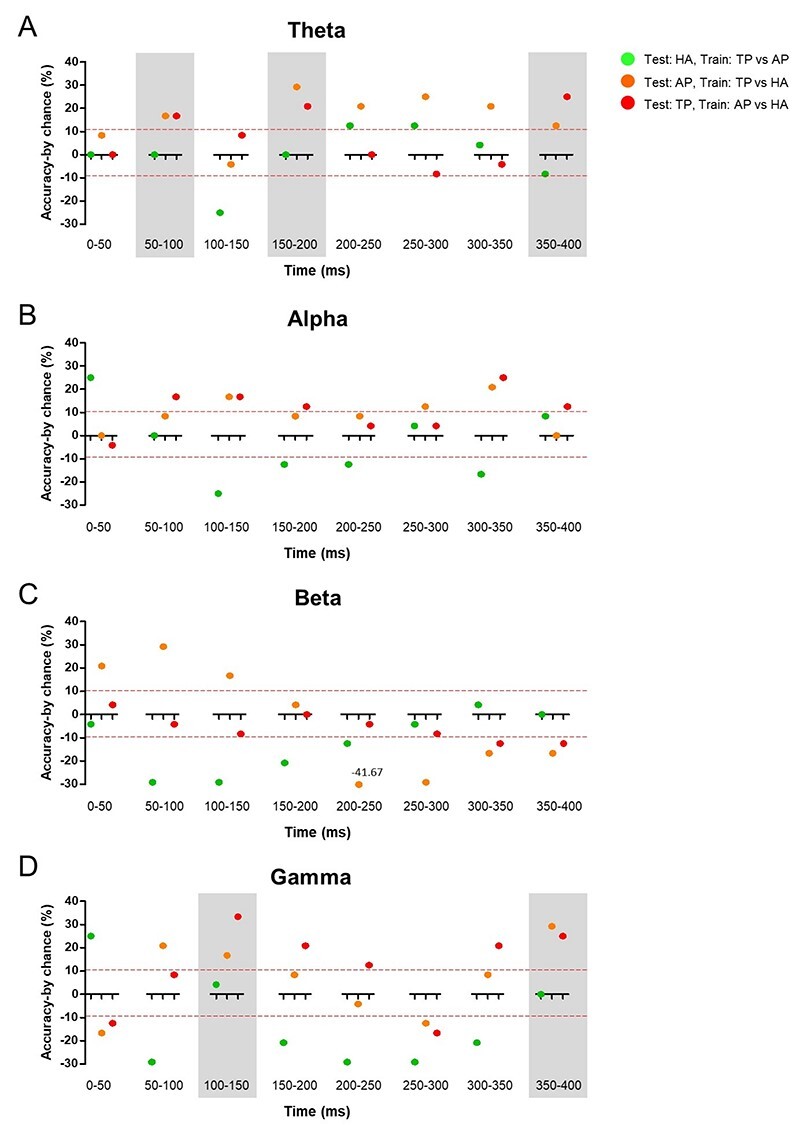
Comparisons of ERSP patterns between odors. ERSP data were vetted using classification analysis. The *y*-axis represents classification accuracy (accuracy by chance), and the *x*-axis represents the time after the onset of odor presentation. Red dashed line represents accuracy by chance = |10%|. Green dots (HA test): positive values indicate that HA was classified as AP; orange dots (AP test): positive values indicate that AP was classified as TP; red dots (TP test): positive values indicate that TP was classified as AP. Gray boxes highlight the results that varied within the same class across the experimental odors.

We found that measurements were > 97% accurate under all conditions. Using the classifiers trained by the ERSPs of two out of the three odor responses, we classified the ERSP induced by the remaining odor that was not used in training to address how ERSP patterns can predict odor similarity or dissimilarity. Across all participants, AP and TP were classified in the same class in theta and gamma (Fig. 3*A* and *D*). Specifically, in the case of theta, AP was classified as TP, and TP was classified as AP with 16.67% accuracy by chance at 50–100 ms. At 150–200 ms, AP was classified as TP with 29.17% accuracy, and TP was classified as AP with 20.83% accuracy by chance. At 350–400 ms, AP was classified as TP with 12.50% accuracy, and TP was classified as AP with 25.00% accuracy by chance. In the case of gamma, AP was classified as TP with 33.33% accuracy, and TP was classified as AP with 16.67% accuracy by chance at 100–150 ms. At 350–400 ms, AP was classified as TP with 25.00% accuracy, and TP was classified as AP with 29.17% accuracy by chance. HA showed about 0% accuracy at these frequencies and time periods (HA accuracy<|10%|).

However, a weak similarity between AP and TP was found at other frequencies. Although AP and TP were classified as the same odors in several time periods of alpha, most time periods showed weak accuracy (below |10%|) or considerable accuracy of HA (over |10%|) (Fig. 3*B*). In the case of beta, AP and TP were not classified as the same, even though there was a weak tendency in all but the 0–50 ms time period. HA and AP were not classified as the same at any frequency, and HA and TP were also not classified as the same except in beta at 250–300 ms. These results indicate that information on odor similarity is contained in the ERSP in several frequency domains with a high temporal resolution.

### Odors Induced Theta and Gamma Activities Relate to Olfactory-Associated Brain Areas at Specific Time Intervals

The above analysis demonstrated that analysis of the theta and gamma ERSP patterns might allow distinction between similar odors at specific time periods. However, further verification of these similarities and their connection to olfactory pathways was necessary. To this end, we estimated the intracerebral electrical sources of theta and gamma frequency bands along the time axis. Using sLORETA, we observed whether time-varying cross-spectra increased significantly between DW and odor stimuli (HA, AP, and TP). We also examined whether different odors elicit differential brain activities, but the patterns were not significantly different among groups (data not shown).

**Figure 4:**
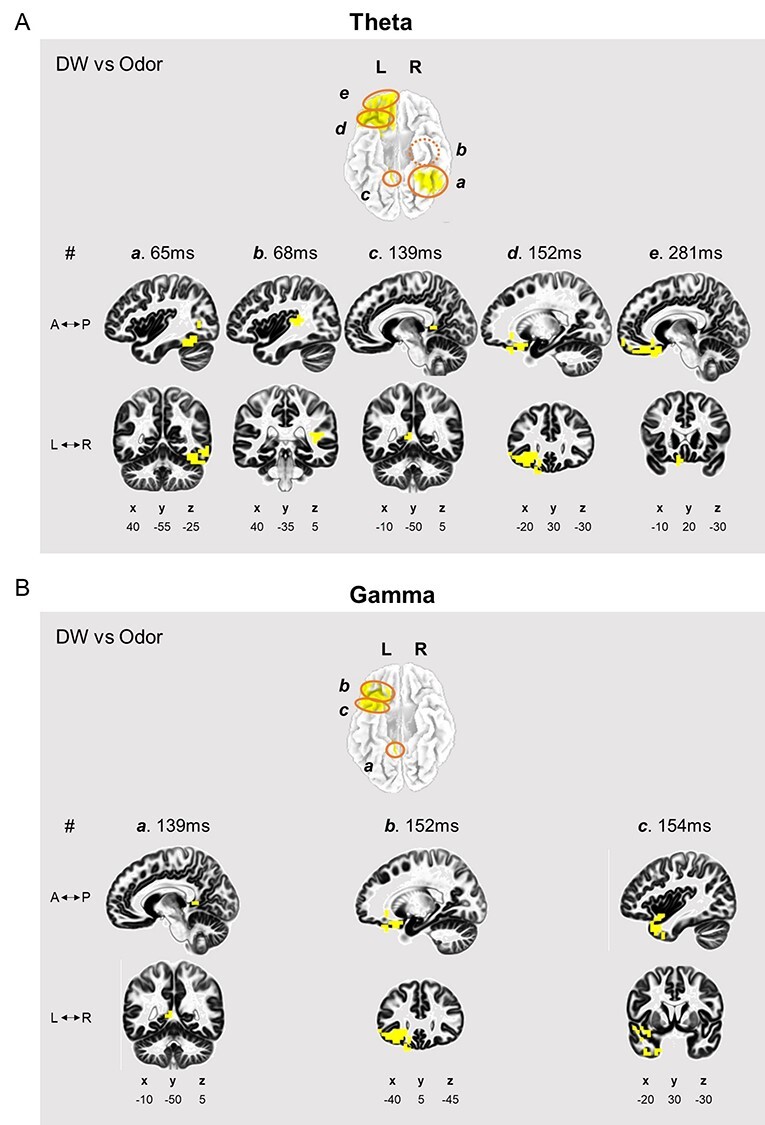
Time-varying activation at theta and gamma frequencies during odor stimulation. Theta (*A*) and gamma (*B*) activity locations estimated by sLORETA. Yellow areas were significantly activated in odor vs. DW comparisons (Bonferroni correction = 0.017).

Figure 4 and Table 1 show significantly activated brain areas in comparisons between DW and odor stimuli (HA, AP, and TP). Within the 0–400 ms time frame, odor conditions (HA, AP, and TP) significantly activated the OFC at theta and gamma frequencies. Specifically, the left middle OFC (BA47) was significantly activated at both frequencies. The left superior OFC was activated only at theta. We found no significant activity in the primary olfactory regions. However, the left posterior cingulate cortex (BA29) located right next to the PC was activated at both frequencies.

**Table 1:** Regions showing significant time-varying activation at theta and gamma frequencies during odor stimulation (Bonferroni correction = 0.017)

Theta			
#	Brain region	Brodmann area	Time (ms)
a	Fusiform gyrus (R)	37	64.45
b	Superior temporal gyrus (R)	41	68.36
c	Posterior cingulate (L)	29	138.67
d	Inferior frontal gyrus (L)	47	152.34
e	Superior frontal gyrus (L)	11	281.25
**Gamma**			
#	Brain region	Brodmann area	Time (ms)
a	Posterior cingulate (L)	29	138.67
b	Superior temporal gyrus (L)	38	152.34
c	Inferior frontal gyrus (L)	47	154.30

Significant theta activity first appeared in the right fusiform gyrus (BA37) at 64.45 ms and almost simultaneously in the right superior temporal gyrus (BA41) (Table 1, upper part). Then, theta activity appeared in the left posterior cingulate cortex (BA29) at 138.67 ms and the left middle OFC at 152.34 ms. Lastly, the left superior OFC activated at 281.25 ms at theta. In the case of gamma, activity first appeared in the left posterior cingulate cortex (BA29) at 138.67 ms, the same time as at theta (Table 1, bottom). Like theta, the left middle OFC was activated at 152.34 ms, but the left superior temporal gyrus (BA38) was also activated at a similar time point, 154.30 ms. Other olfactory-associated areas were activated at 60, 100–150, and 350–400 ms in theta (Supplementary Table 1) and at 40, 150, and 350–400 ms (Supplementary Table 2), although these activation events were marginally significant.

## Discussion

The primary aim of this study was to outline when the brain categorizes odor by object quality during olfactory processing. Using similar odors, AP and TP, and a distinct odor, HA, we focused on when and how pairs of these odors induced a similar neural cascade. We first verified similarity across the experimental odors. As expected, we found that AP and TP showed similar object quality and were distinct from HA (Fig. 2). Next, we found that AP and TP induced similar multivariate ERSP patterns at theta and gamma frequencies (Fig. 3*A* and *D*). In SVM analysis, AP and TP induced similar theta waves at 100, 150–200, and 350–400 ms. Gamma also showed similar AP and TP activation at 100–150 and 350–400 ms. These neuronal activities were related to olfactory pathways. We found that theta and gamma activities were related to olfactory-associated brain areas during odor stimulation (Fig. 4 and Table 1, Supplementary Tables 1 and 2). The middle and superior OFC were significantly activated, and primary olfactory cortex regions were also activated. Moreover, these sLORETA results also showed similar time intervals to Figure 3. Although we observed no differences among the experimented odors, we found that olfactory-associated areas were activated at 50–200 and 350–400 ms, time intervals similar to those suggested in Figure 3. These results suggest that odor information may be encoded at specific time periods at theta and gamma frequencies, and that olfactory-associated areas may be strongly involved in these specific periods.

According to our results, at least two separate periods may exist during odor quality processing in our brain (Figs 3 and 4). AP and TP were classified as the same odor in three separate periods at theta and in two separate periods at gamma. This means that odor categorization may consist of several or at least two separate steps before it is expressed by behavior. We found that 50–200 and 350–400 ms may be the critical time periods after odor stimulation. Although the significance was only marginal, primary olfactory areas were activated at 60–150 ms, and OFC was significantly activated at 150–200 ms in the first neural cascade (Table 1, Supplementary Tables 1 and 2). Interestingly, a second neural cascade was observed around 350 ms, i.e., 150 ms after the first cascade. In this period, we found that not only secondary but also primary olfactory areas were activated, along with the amygdala, entorhinal cortex, and hippocampus (Supplementary Tables 1 and 2). This activation of multiple olfactory-associated areas during processing is also in line with an MEG study that showed that olfactory-associated areas were activated at least twice during odor stimulation (Stadlbauer et al. 2016). However, our results further suggest that olfactory processing occurs through the activation of multiple brain regions in at least two separate time windows.

We lack evidence to interpret each period, but according to previous studies, we can hypothesize that the first period may be the information processing step, while the second may be the integration step (Bar et al. 2006; Hegdé 2008). In the visual system, early OFC activity (130 ms) is sensitive to spatial frequencies rather than the integration role of sensory or preceding information (Bar et al. 2006; Hegdé 2008). Although this evidence is from another sensory system, it suggests that the OFC processes primary odor information mainly at an early time point (~150 ms). However, to produce proper responses, an integration step is needed. In the olfactory system, more than 300 ms may be necessary for proper odor responses. In rodent studies, ~300 ms was the limit of deliberation for proper odor discrimination (Uchida and Mainen 2003; Zariwala et al. 2013). It takes humans at least 400 ms to discriminate odors (Laing 1986; Olofsson et al. 2013). These lines of evidence imply that sufficient odor responses require more than 300 ms. Thus, the second period may correspond to the integration step for proper odor responses.

The time course of the activation of olfactory-associated areas in our study was similar to those in EEG-fMRI and MEG studies. Masaoka et al. (2014) reported that the primary olfactory area (PC, part of the entorhinal cortex, and amygdala) exhibits very early activation (around 50 ms after stimulus presentation), which is similar to the early activation in the OFC that we observed here. Moreover, their findings of a weak signal from the PC and a tendency for left hemisphere dominance align with our results. A MEG study by Stadlbauer et al. (2016) suggested a rather late activation of the primary olfactory area (~80 ms) but similar activation in the OFC. They also suggested two or more periods of activation of the olfactory-associated areas over 200 ms.

However, some of our results differ from those of the previous studies. We found early activation in the fusiform gyrus and superior temporal gyrus (at 64–70 ms). These results are counterintuitive because these areas are well known for facial and auditory processing rather than olfactory processing. Functional connectivity studies suggest a clue to reconcile this discrepancy. According to Zhou et al. (2019), these two areas are functionally connected with the olfactory-associated area. The fusiform gyrus is functionally connected with the anterior olfactory tubercle and the frontal and temporal PC, whereas the superior temporal gyrus is connected with the temporal PC. Moreover, the right fusiform gyrus may be involved in odor recognition during olfactory processing (Zhou et al. 2019). In an experiment with early-blind subjects by Renier et al. (2013), the right fusiform gyrus was activated during odor stimulation, and its activation correlated with odor recognition test results. The superior temporal gyrus is also related to the olfactory system (Zhou et al. 2019). Few studies are available on the olfactory system’s connection with the fusiform gyrus and superior temporal gyrus, and more studies are needed in the future. Additionally, we found odd olfactory processing patterns, especially in the PC (Supplementary Table 1). Although other primary olfactory cortex, such as the entorhinal cortex, activated faster than the PC, it activated similar to secondary olfactory cortexes. This may be the result of a different pattern from that used in previous studies because the PC is anatomically part of the primary olfactory circuit. Moreover, the PC showed insufficient activity even though it is well known that it plays an essential a role in the olfactory process. One possible explanation is the transient response of the PC activation. Noam Sobel et al. (2000) showed PC transient responses to odorants, and Andreas Stadlbauer et al. (2016) also showed a similar pattern by MEG (Sobel et al. 2000; Stadlbauer et al. 2016). This evidence was in line with several previous studies that failed to observe significant activation during olfaction, especially in initial fMRI and PET studies (Yousem et al. 1997; Dade et al. 1998; Sobel et al. 1998). However, this explanation is linked with a methodological problem because MEG and intracortical EEG studies showed early activation of the PC (~100 ms) during olfaction. (Stadlbauer et al. 2016; Jiang et al. 2017). Since we used scalp EEG and estimated EEG sources by sLORETA, a false negative may have increased in our experimental circumstance compared to other methodological studies.

Interestingly, theta and gamma waves were the most represented frequency bands, which implies that they may play a role in olfactory processing. We found that, although activation at these two frequency bands is detectable in the same areas, the activation occurs at different time points when they represent odor quality (Fig. 3). This difference in time points may be caused by the characteristics of the frequency bands, namely that the higher frequency is hard to detect from deep brain structures. Thus, the location of primary olfactory areas deep in the brain may reduce the sensitivity. However, in a previous study that used intracortical EEG, theta waves were induced by odor stimulation in the PC, suggesting that theta waves may originate from odor processing rather than other frequencies (Jiang et al. 2017), and gamma may represent olfactory processing specifically in the OB (Iravani et al. 2020). This means that although theta and gamma activated in many consensus areas, the origin of these two frequencies may be different; rodent studies have also suggested different roles of these frequencies (Adrian 1942; Bressler 1984; Vanderwolf 1992; Chapman et al. 1998; Kay and Freeman 1998; Mori et al. 2013).

The following points should be considered when interpreting our results. While SVM is a valuable analysis tool for handling multivariate data, it does not give us detailed results. Thus, our SVM results may be less relevant than our sLORETA results. Because we had no significant differences in results among the experimental odors in sLORETA analysis, interpreting the relation of SVM and sLORETA results should be carefully considered. However, we focused on outlining odor quality processing, and our results suggest which frequency is involved during which time period while processing is underway. Moreover, our spatial analysis showed significant PC and OFC activation, highly related to odor processing. Thus, we assume that olfactory-associated areas are involved in SVM results. Secondly, sLORETA results should be carefully interpreted because this method suggests estimating intracerebral electrical sources, not the actual activities. Additionally, olfactory-associated brain areas are located in the deep brain structure, so the error rate may be higher than that in other brain areas. Indeed, previous studies suggested evidence of proper estimation of deep brain structures (Zumsteg et al. 2006; Sohrabpour et al. 2015; Iravani et al. 2020), but there are limited studies of the primary olfactory cortex. Moreover, few studies focused on the relationship between signal intensity and sLORETA accuracy in the deep brain areas, so a weak EEG signal may be hard to cover by sLORETA. Lastly, we have to consider trigeminal activation by odorants. Because our experimental procedure conveys odor to nose inspiration, it can induce trigeminal activation. Our results may have consisted of trigeminal input and as such may vary in the different experimental procedures that controlled trigeminal activation.

## Conclusion

Here, we provide essential evidence concerning when the brain differentiates among odors at an unprecedented temporal resolution during the olfactory process. Although previous studies also provide information on the temporal activities of the brain during odor stimulation, those have not been clearly verified during odor information processing. In our study, we verified when and how odor information could be categorized during processing. Odors that are perceived as similar induce similar theta and gamma patterns of brain activity, and these patterns occur during a specific period. These patterns are significant in olfactory-associated areas, where theta and gamma are strongly correlated with odor information processing in humans.

## Supplementary Material

Supplementary_Material_tgab058
