# Supplementary material for: Time Course of Odor Categorization Processing
Source: Cereb Cortex Commun. 2021 Oct 5;2(4):tgab058. doi: 10.1093/texcom/tgab058 (PMC8567848; doi:10.1093/texcom/tgab058)
Supplement: Supplementary_Material_tgab058 [file supplementary_material_tgab058.docx]

**Supplementary Figures**


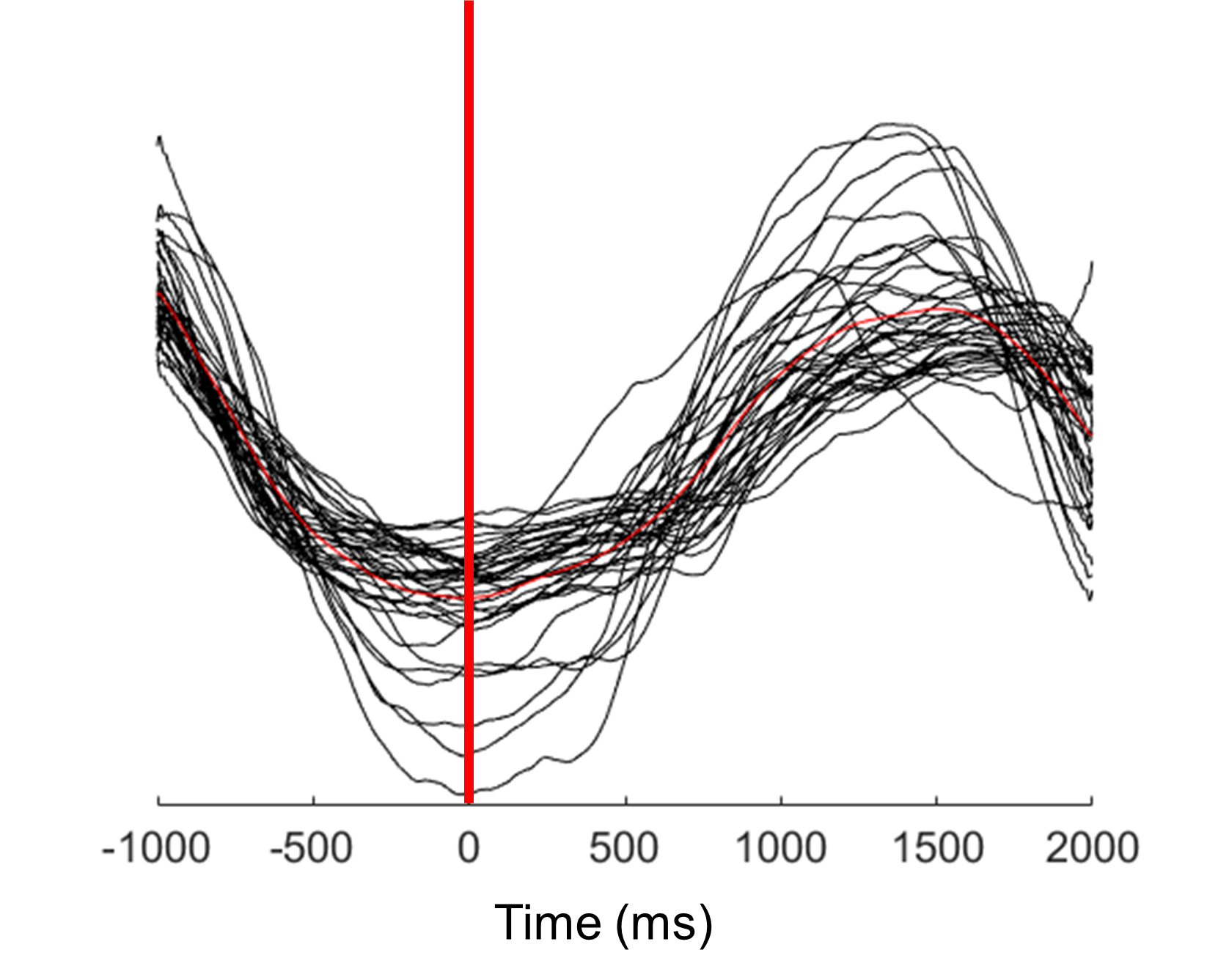


S Figure. 1 Inspiration staring point. A representative respiration cycle is shown. EEG epochs were time-locked at the inspiration starting point (0 sec). The red line is the average of the respiration of the whole epochs.


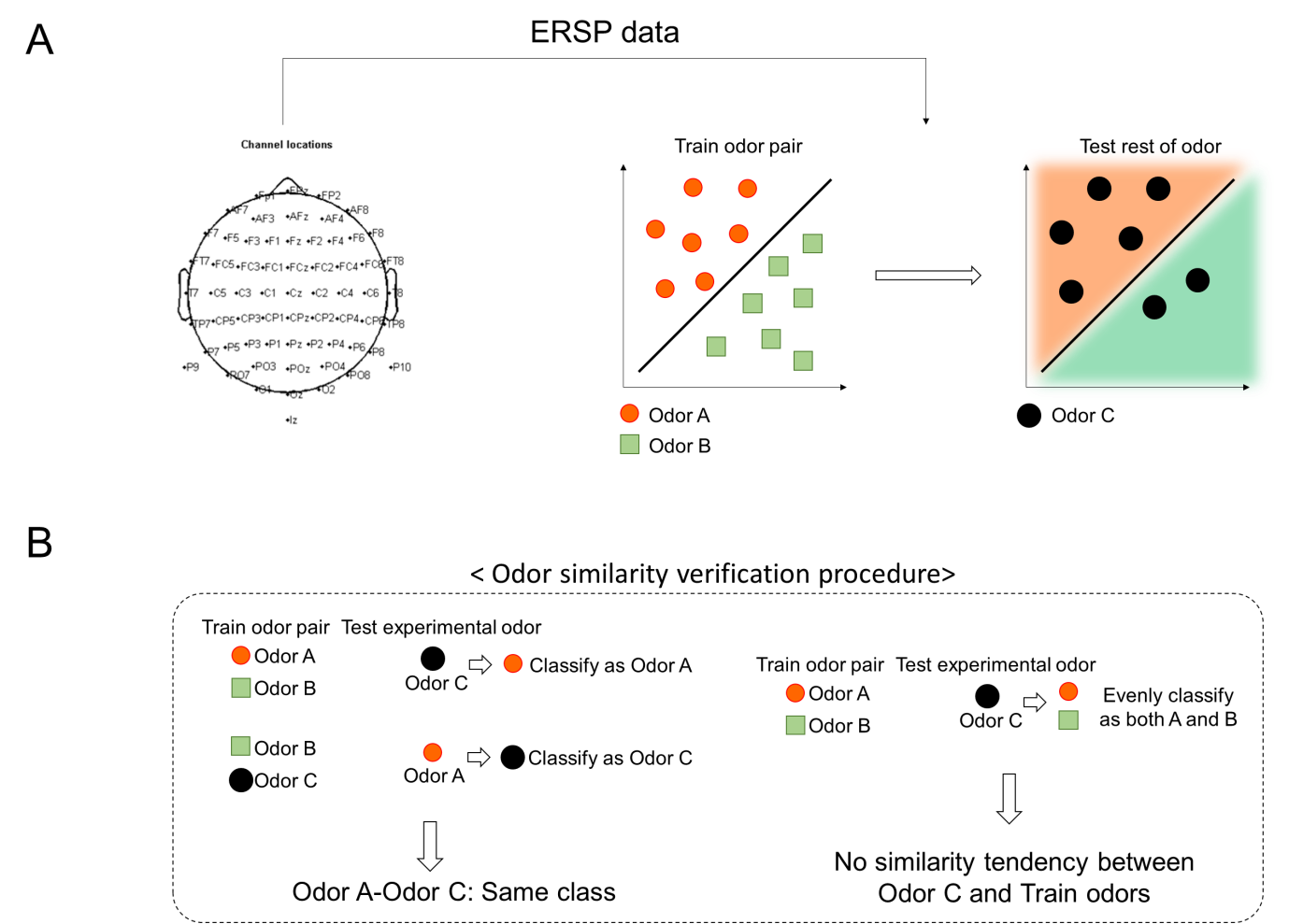


S Figure. 2 Verifying the pattern of similarity by classification analysis. A. Classification procedure designed to verify ensemble-pattern coding of odor category data. ERSP data from each odor were vetted during classification analysis. AP vs. TP, HA vs. AP, or HA vs. TP were used as training datasets. HA, TP, or AP, respectively, were used as test datasets. First, a linear SVM was trained using training datasets to classify two odors (A, middle panel). Second, test datasets were used to test how well the model can distinguish between two distinct odors classified as similar (A, right panel). B. Odor similarity verification procedure. When two odors were classified as the same by the procedure outlined in A, these odors were paired in the same class. When the odor was not classified as similar to either of the training odors in a pair, the odor was identified as having no similarity to the training odors.


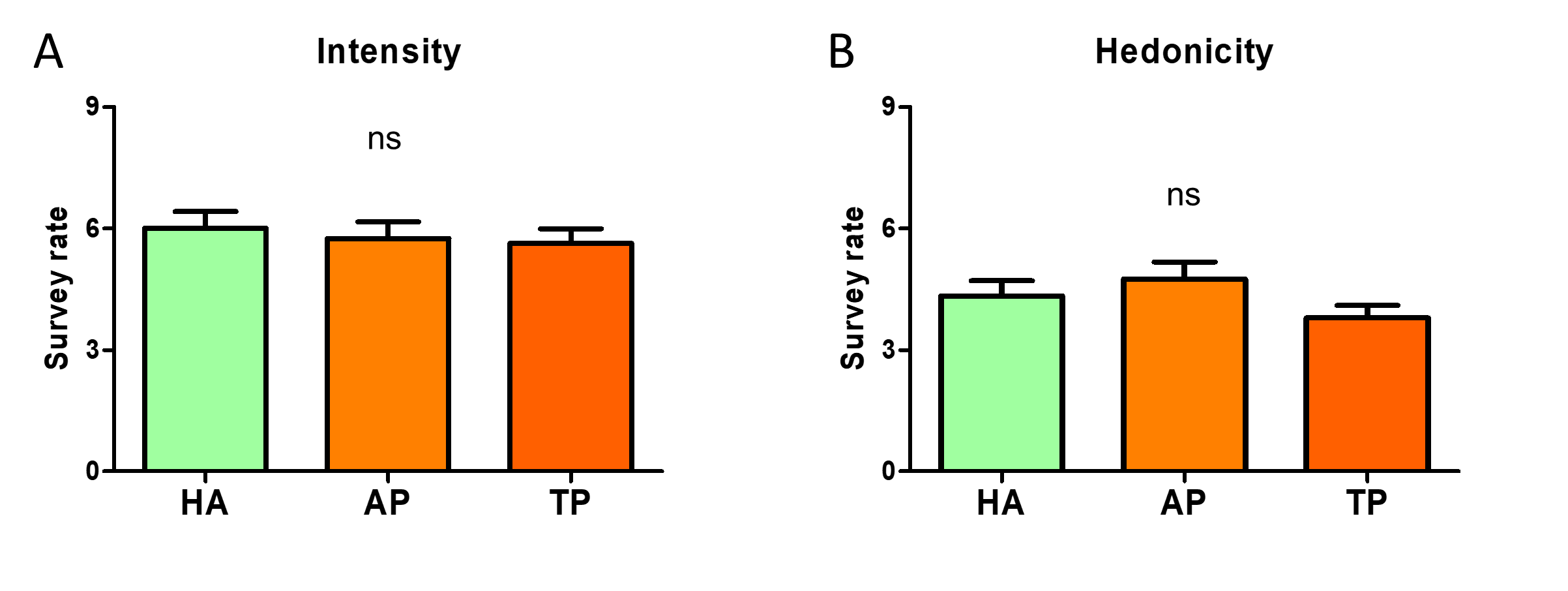


S Figure. 3 Intensity and hedonicity of odors. There were no significant differences among odors.

**Supplementary Tables**

| **Theta** | | | | |
| --- | --- | --- | --- | --- |
|  | **Brain region** | **BA** | **First appearance (ms)** | **Second appearance (ms)** |
| **Left hemisphere** | Ventral entorhinal cortex | 28 | 60.55 | 378.91 |
|  | Middle orbitofrontal cortex | 47 | 152.34 | . |
|  | Superior orbitofrontal cortex | 11 | 279.30 | . |
|  | Part of the perirhinal cortex  (including the hippocampus, laterally by the BA 36) | 35 | 357.42 | 378.91 |
|  | Subgenual area (including the amygdala) | 25 | 376.95 | . |
|  | Dorsal entorhinal cortex | 34 | 378.91 | . |
|  | Part of the perirhinal cortex  (including the hippocampus, medially by the BA 35) | 36 | 378.91 | . |
| **Right hemisphere** | Piriform cortex | 27 | 138.67 | . |
|  | Subgenual area  (including amygdala) | 25 | 140.63 | 376.95 |
|  | Part of the perirhinal cortex  (including the hippocampus, laterally by the BA 36) | 35 | 140.63 | . |

S Table. 1 Theta activities in olfactory-associated areas (*p* <0.05, not Bonferroni corrected)

| **Gamma** | | | | |
| --- | --- | --- | --- | --- |
|  | **Brain region** | **BA** | **First appearance (ms)** | **Second appearance (ms)** |
| **Left hemisphere** | Middle orbitofrontal cortex | 47 | 152.34 | . |
|  | Superior orbitofrontal cortex | 11 | 279.30 | . |
|  | Ventral entorhinal cortex | 28 | 378.91 | . |
|  | Dorsal entorhinal cortex | 34 | 378.91 | . |
|  | Part of the perirhinal cortex  (including the hippocampus, laterally by the BA 36) | 35 | 378.91 | . |
|  | Part of the perirhinal cortex  (including the hippocampus, medially by the BA 35) | 36 | 378.91 | . |
| **Right hemisphere** | Dorsal entorhinal cortex | 34 | 42.97 | . |

S Table. 2 Gamma activities in olfactory-associated areas (*p* <0.05, not Bonferroni corrected)
